# CRISPR/Cas9‐guided knockout of *eIF4E* improves *Wheat yellow mosaic virus* resistance without yield penalty

**DOI:** 10.1111/pbi.14002

**Published:** 2023-01-24

**Authors:** Jinhong Kan, Yu Cai, Chunyuan Cheng, Shiqiang Chen, Congcong Jiang, Zhentian He, Ping Yang

**Affiliations:** ^1^ Institute of Crop Sciences Chinese Academy of Agricultural Sciences (CAAS) Beijing China; ^2^ Agricultural Sciences Institute in Jiangsu Lixiahe Area Yangzhou China

**Keywords:** wheat (*Triticum aestivum*), barley (*Hordeum vulgare*), *eIF4E*, host factor, genetic resistance, genome editing

Viruses, one of the most agriculturally important groups of plant pathogens, account for 47% of all emerging plant infectious diseases (Anderson *et al*., 2004). Viruses with relatively simple genomes and encoding only a few proteins are highly dependent on host factors (or susceptibility [S] factors) to complete their lifecycles. The modification or mutation of S factors might cause the loss of susceptibility, often resulting in recessive resistance. The *eukaryotic translation initiation factor 4 E* (*eIF4E*) is a well‐characterized *S* gene for many potyviruses of the family *Potyviridae* in dicots, which encodes a cap‐binding protein that binds to methylated guanine triggering assembly of the protein translation initiation complex. The *eIF4E* has become a major target for engineering viral resistance using the CRISPR/Cas9 (clustered regularly interspaced short palindromic repeats/CRISPR‐associated protein 9) genome‐editing technology. Knocking out *eIF4E* was shown to result in resistance in dicots such as melon (Pechar *et al*., 2022) and monocots such as barley (Hoffie *et al*., 2021).

Wheat yellow mosaic disease caused by *Wheat yellow mosaic virus* (WYMV) or *Chinese wheat mosaic virus* (CWMV) in East Asia, and *Wheat spindle streak mosaic virus* (WSSMV) in Europe and North America, results in severe yield losses in hexaploid wheat. WYMV and WSSMV belong to the genus *Bymovirus* of the family *Potyviridae* and are transmitted by the soil‐borne plasmodiophorid *Polymyxa graminis*. In diploid barley, the bymoviruses *Barley yellow mosaic virus* (BaYMV) and *Barley mild mosaic virus* (BaMMV) cause yellow mosaic disease, and most resistance loci are represented by recessive alleles (Jiang *et al*., 2020). By contrast, 13 resistance genes and/or quantitative trait loci (QTL) have been mapped in wheat, and all are inherited in a dominant manner. However, to date, no resistance genes against viruses have been isolated by map‐based cloning in wheat.

This study reported CRISPR/Cas9‐guided genome editing of wheat orthologous of the barley susceptibility factor gene *HveIF4E*. In barley, natural polymorphisms in *HveIF4E* are the causal agent of several resistance alleles (Jiang *et al*., 2020), and *HveIF4E*‐edited plants also showed BaMMV resistance (Hoffie *et al*., 2021). Three *TaeIF4E* homoeologous genes were identified by BLAST against the reference genome sequence of wheat (IWGSC RefSeq v1.1; Figure S1a). We selected a 20‐bp target sequence for a single guide RNA (sgRNA) upstream of the protospacer‐adjacent motif (PAM) within the conserved region in these three homoeoalleles (Figure 1a and Figure S1a,b) and transformed the expression cassette into wheat cultivar (cv.) ‘Fielder’. Out of 65 independent regenerated plants (T_0_), eight genome‐editing events were detected (Table S1). Seven of eight edits were single base insertions or deletions of 1 or 2 bp leading to frameshift mutations. There were three, three, and two plants showing edits at the target site in subgenomes 3A, 3B, and 3D, respectively (Table S1). We conducted two rounds of cross‐pollination and marker‐assisted selection to pyramid the *TaeIF4E* mutations in all three subgenomes in a single hybrid plant, followed by genotyping 318 F_2_ segregants (Figure S1b–e). Single (*aaBBDD*, *AAbbDD* and *AABBdd*), double (*aabbDD*, *aaBBdd* and *AAbbdd*) and triple (*aabbdd*) mutants in all three *TaeIF4E* homoeoalleles were obtained. The CRISPR/Cas9 cassette was present in all mutants (Figure S1f). We predicted and analysed five potential off‐target effects, and no mutations were detected in mutants (Table S2), indicating off‐targets unlikely occurred.

**Figure 1:**
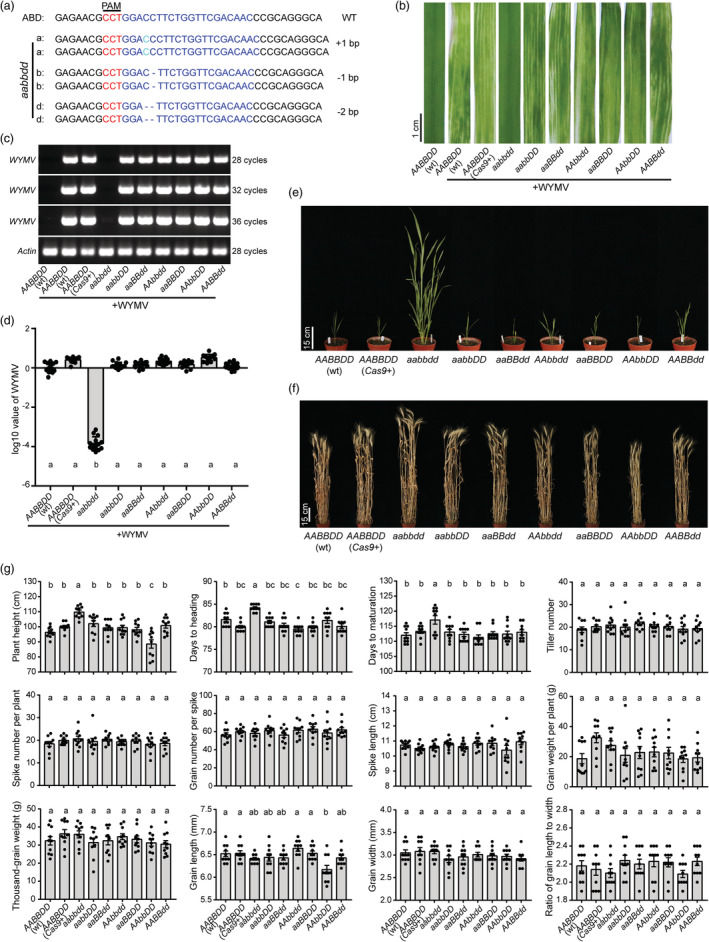
*TaeIF4E*‐edited triple mutant improves WYMV resistance without yield penalty in wheat. (a) Mutations in the *TaeIF4E* nucleotide sequence in triple mutants. The sgRNA and the protospacer‐adjacent motif (PAM) are highlighted in blue and red, respectively. WT, wild type. (b) The symptoms of the triple, double and single mutants, the wild type (wt, non‐transformed ‘Fielder’) and the mock control (harbouring the *Cas9* cassette, without detected edits) at 6 weeks postinoculation with WYMV. The accumulation of WYMV was determined by RT‐PCR (c) and RT‐qPCR (d). The wheat *Actin* gene served as the endogenous control. In (d), black dots represent samples (*n* = 14). (e) Phenotypes of the *TaeIF4E*‐edited mutant lines at jointing stage under greenhouse conditions. Eight‐week‐old WYMV‐inoculated plants were transplanted to soil and grown in the greenhouse conditions until seed harvest. (f) Phenotype of *TaEIF4E‐*edited mutants at the maturation stage in garden experiment. (g) Statistics for 12 agronomic traits. Ten plants per genotype were analysed. Black dots represent individual measurements. Statistical significance was determined using Tukey's *post hoc* test (*P* = 0.05). Error bars represent standard deviation (SD).

We mechanically inoculated nine homozygous lines, representing edited mutants, non‐edited plants (*AABBDD*/*Cas9*+) and wild‐type ‘Fielder’ with WYMV‐infected wheat leaves or mock (buffer). The mock‐inoculated plants stayed green and grew normally (Figure S2a,b). However, upon WYMV infection, only the triple‐edited plants remained green, resembling wild‐type plants without infection, whereas the other plants were susceptible to WYMV with yellow discoloration (Figure 1b). By using reverse transcription (RT)‐PCR and quantitative RT‐PCR, viral RNA accumulation was hardly (ca. 10^−4^) detected in triple‐edited plants, whereas abundant viral RNA accumulated in wild‐type plants and other mutants (Figure 1c,d). We transplanted 8‐week‐old plants that had been inoculated with WYMV or mock to the normal greenhouse conditions. Whereas triple‐edited plants that were inoculated with WYMV grew normally, plants of the other genotypes showed severely stunted growth (Figure 1e), fewer spikes and lower seed setting (Figure S3a). Both mock‐inoculated *TaeIF4E*‐edited mutants and wild‐type plants were well developed (Figure S3b). In addition, we inoculated these genotypes with CWMV, another soil‐borne *P. graminis*‐transmitted virus that belongs to the genus *Furovirus* of the *Virgaviridae* family. All the *TaeIF4E*‐edited mutants were susceptible to CWMV, like wild‐type ‘Fielder’, with yellow chlorotic streaking or stripes on the leaves (Figure S4a). We observed no significant differences in viral accumulation among lines (Figure S4b,c). These results demonstrate that TaeIF4E is a host factor in the host compatibility of WYMV but not CWMV.

We further recorded 12 agronomic traits under normal growth conditions in garden experiment. The *TaeIF4E‐aabbdd* mutant showed an elevated plant height, as these plants were ca. 10 cm taller than the other lines (Figure 1f,g), as well as delayed heading (ca. 4 days) and maturity (ca. 5 days; Figure 1g and Figure S5). Except the single mutant *TaeIF4E‐AAbbDD* that showed a significant difference in plant height and grain length, no significant differences were detected for the remaining traits (Figure 1g). The simultaneous targeting of the three *TaeIF4E* homoeoalleles resulted in pleiotropic changes in plant height and heading/maturity, but without yield penalty.

Collectively, our results demonstrate that CRISPR/Cas9‐guided knockout of *TaeIF4E* improved WYMV resistance in hexaploid wheat. This is identical in principal to our finding that the simultaneous editing of *TaPDIL5‐1* homoeoalleles conferred complete resistance against WYMV (Kan *et al*., 2022). The triple‐mutated *TaeIF4E* (this study) or *TaPDIL5‐1* (Kan *et al*., 2022) plants were resistant against WYMV, whereas the single or double‐mutants were susceptible, demonstrating the functional redundancy of the homoeoalleles of the host factor genes and their ability to compensate for each other to facilitate WYMV infection. These results explain the recessive resistance to bymoviruses that has often been detected in diploid barley but not in polyploid wheat (Jiang *et al*., 2020). Importantly, we detected no yield penalty in the *TaeIF4E* triple‐edited mutant. This observation is different from diploid barley, in which yield reduction was observed (Hoffie *et al*., 2021) and in diploid tomato, with severe dwarfing or growth defects (Gauffier *et al*., 2016) as well as diploid melon, with male sterility (Pechar *et al*., 2022). We speculate that there are unknown mechanisms that complement with the *eIF4E* deficiency in polyploidy wheat. Our results highlight the potential of engineering virus resistance via genome editing of *eIF4E* in polyploid species such as hexaploid wheat.

## Conflict of interest

A patent application related to this work has been filed.

## Author contributions

PY designed the research. JK, YC, CC, PY, CJ, SC and ZH performed the experiments and data analysis. PY and JK wrote the manuscript. All authors read and approved the final manuscript.

## Supporting information


**Table S1.** Editing events in 65 regenerated T_0_ plants, as determined by Sanger sequencing of plasmids derived from PCR products.
**Table S2.** Analysis of potential off‐target effects in edited mutants.
**Table S3.** PCR primers used in this study.

**Figure S1.** Generation of *TaeIF4E*‐edited mutants by genome editing and marker‐assisted allele stacking.

**Figure S2.** Phenotypes of the *TaeIF4E*‐edited mutant lines following mock inoculation under greenhouse conditions.

**Figure S3.** Phenotypes of WYMV‐inoculated and mock‐inoculated plants at the maturation stage under greenhouse conditions.

**Figure S4.** *TaeIF4E*‐edited triple mutant in common wheat shows no resistance to CWMV.

**Figure S5.** Triple knockout *TaeIF4E* mutant shows delayed maturation.
