# Supplementary material for: CRISPR/Cas9‐guided knockout of eIF4E improves Wheat yellow mosaic virus resistance without yield penalty
Source: Plant Biotechnol J. 2023 Jan 24;21(5):893–5. doi: 10.1111/pbi.14002 (PMC10106853; doi:10.1111/pbi.14002)
Supplement: Supplementary file 1 — Table S1. Editing events in 65 regenerated T0 plants, as determined by Sanger sequencing of plasmids derived from PCR products. Table S2. Analysis of potential off‐target effects in edited mutants. Table S3. PCR primers used in this study. [file PBI-21-893-s003.docx]

**Supporting Information for**

**CRISPR/Cas9-guided knockout of *eIF4E* improves *Wheat yellow mosaic virus* resistance without yield penalty**

1. **The five supplementary Figures are available for this article:**

**
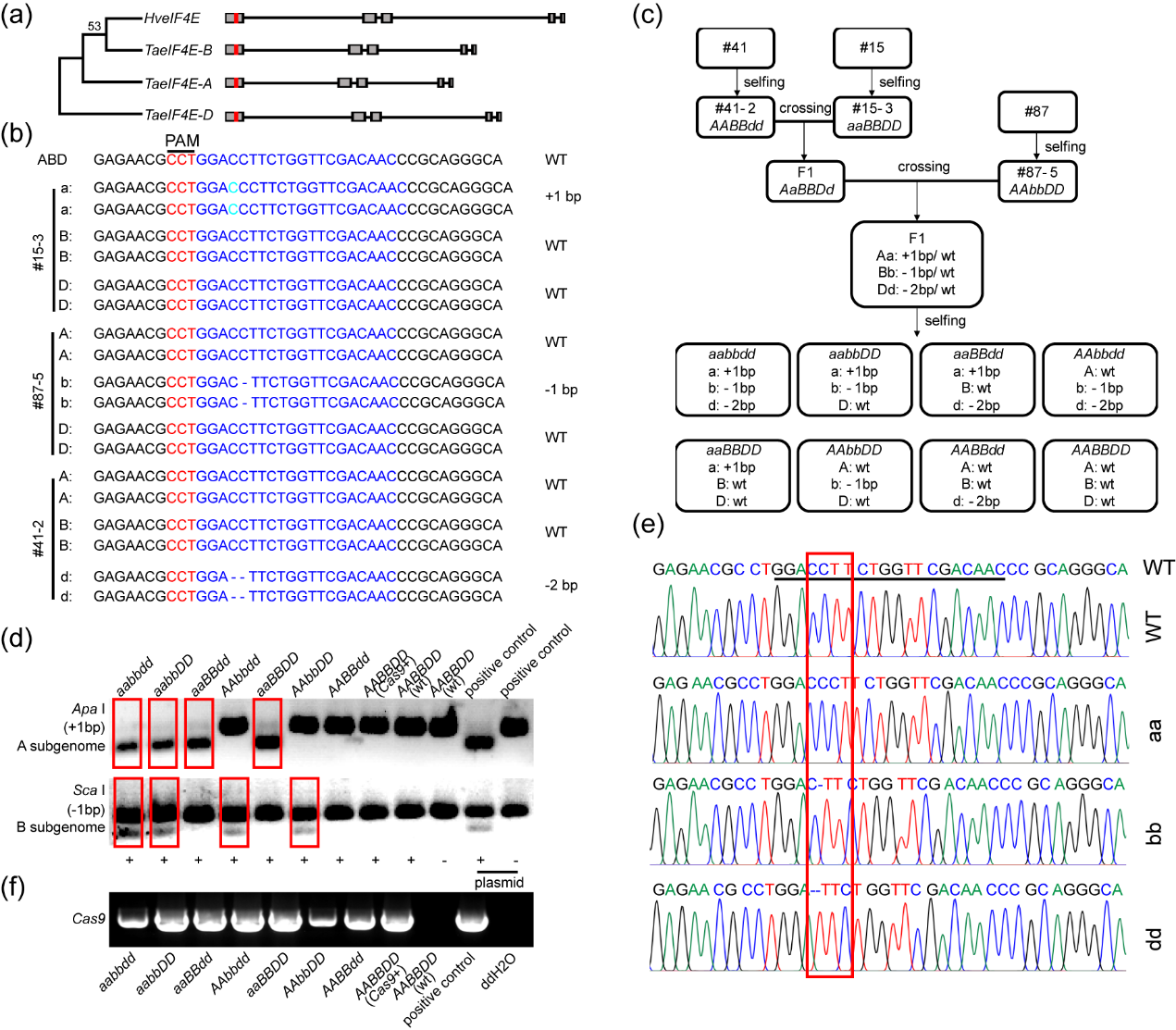
**

**Figure S1**. Generation of *TaeIF4E*-edited mutants by genome editing and marker-assisted allele stacking. (a) Schematic representation of wheat *TaeIF4E* homoeoalleles and their ortholog *HveIF4E* in barley. *TaeIF4E-A*, TraesCS3A02G521500.1; *TaeIF4E-B*, TraesCS3B02G591300.1; *TaeIF4E-D*, TraesCS3D02G527800.1. The unrooted phylogenetic tree was produced by the neighbor-jointing algorithm using the coding sequences of each gene (bootstraps = 1,000). The target site for the sgRNA within a conserved region at the first exon in the *TaeIF4E* homeologs is highlighted in red. Exons and introns are shown as gray boxes and black lines, respectively. (b) Changes in the *TaeIF4E* nucleotide sequence in three single mutants. The sgRNA and the protospacer adjacent motif (PAM) are highlighted in blue and red, respectively. WT, wild type. (c) Workflow for pyramiding *TaeIF4E* frameshift alleles in the three wheat subgenomes. (d) Genotyping using allele-specific dCAPS markers. The mutated allele for each subgenome is highlighted by a red rectangle. ‘+’ and ‘–’ indicate digestion reactions with and without restriction enzymes, respectively. PCR products amplified from plasmid template were used as the control. The mutated allele in the D subgenome was genotyped by Sanger sequencing and is not shown in the figure. (e) Sanger sequencing chromatograms of the target sites in the three subgenomes of the *TaeIF4E*-edited triple mutant. (f) PCR amplification of the CRISPR/Cas9 cassette.


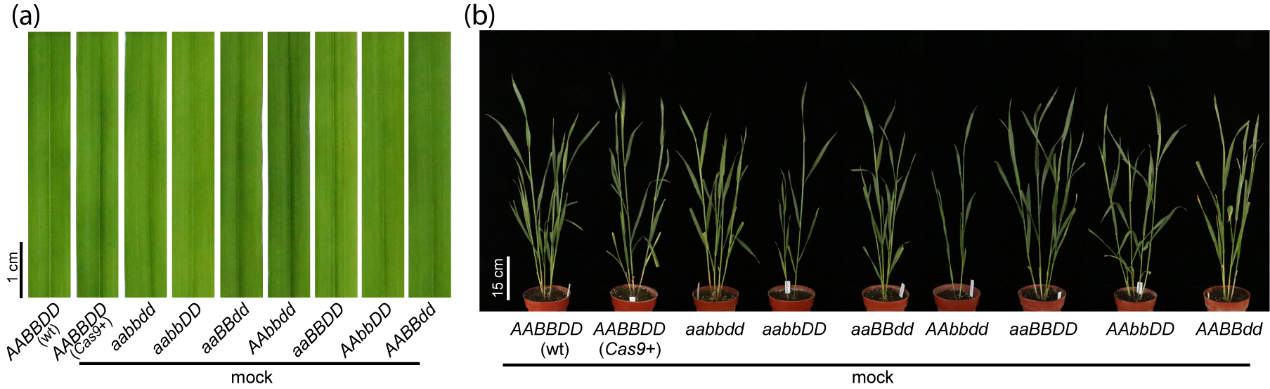


**Figure S2**. Phenotypes of the *TaeIF4E*-edited mutant lines following mock inoculation at seedling stage and jointing stage under greenhouse conditions. (a) The leaves of the *TaeIF4E* mutant lines at seedling stage, and the photographs were taken at six weeks post mock inoculation. (b) Phenotypes of the *TaeIF4E*-edited mutant lines following mock inoculation at jointing stage under greenhouse conditions. Eight-week-old mock-inoculated plants were transplanted to soil and grown in the greenhouse conditions until seed harvest.


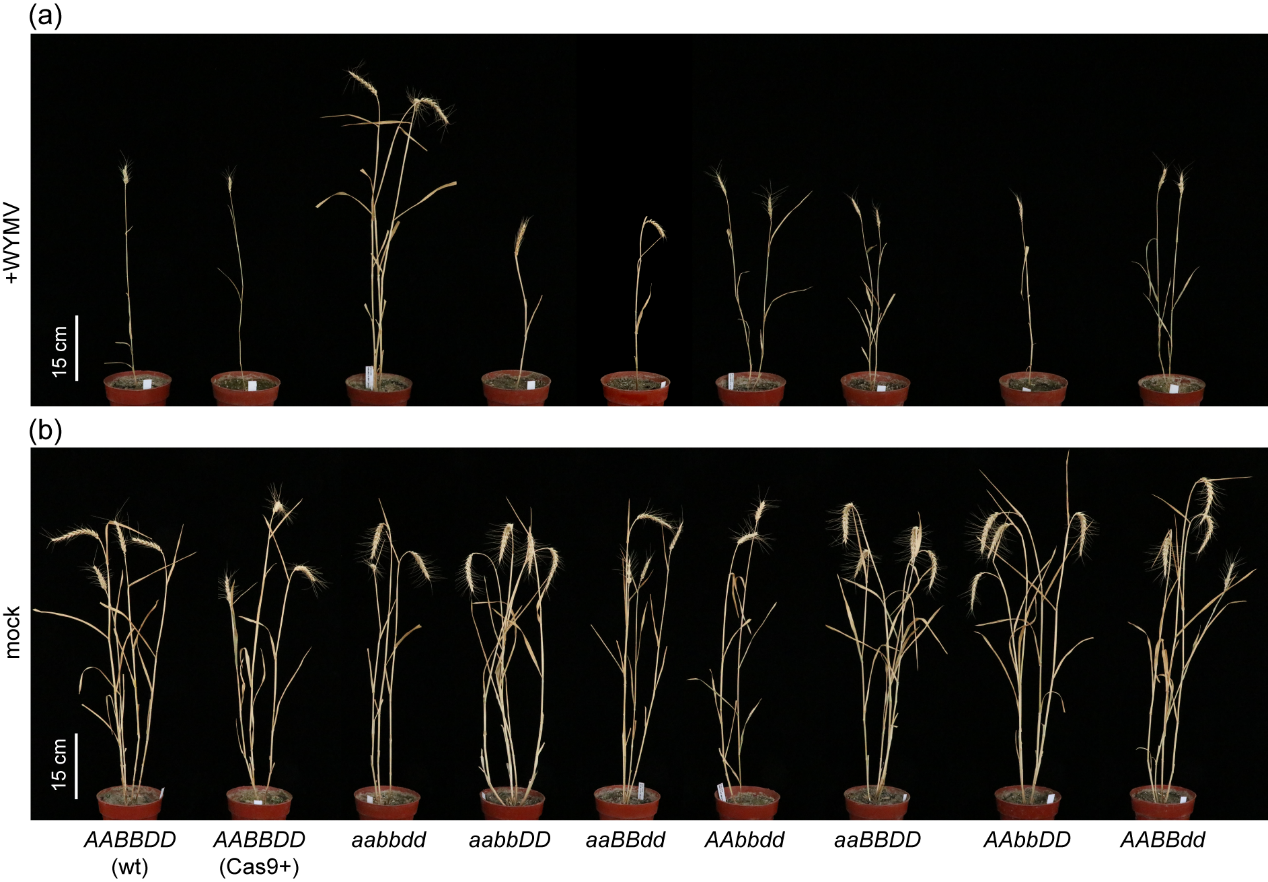


**Figure S3.** Phenotypes of WYMV-inoculated (a) and mock-inoculated plants (b) at the maturation stage under greenhouse conditions. Eight-week-old mock-inoculated plants were transplanted to soil and grown in the greenhouse conditions until seed harvest. Whereas WYMV-inoculated triple mutant plants grew normally, like mock-inoculated plants, all other plants showed dwarfism, poor seed setting, and severe yield reduction following WYMV inoculation.

**
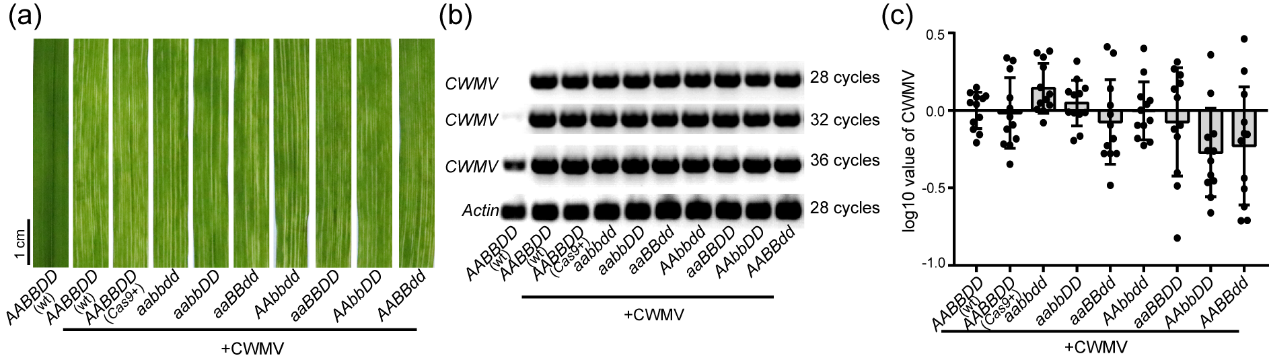
**

**Figure S4**. The *TaeIF4E*-edited triple mutant in common wheat shows no resistance to CWMV. The symptoms of the triple, double, single mutants, the wild type (wt, non-transformed Fielder), and the mock control (harboring the *Cas9* cassette, without detected edits) were captured at six weeks post inoculation with CWMV (a). The accumulation of CWMV was quantified by RT-PCR (b) and RT-qPCR (c). The wheat *Actin* gene served as the endogenous control. In (c), black dots represent samples (n = 12). Statistical significance was determined by analysis of variance (ANOVA) followed by Tukey’s *post hoc* test (*p* = 0.05). Error bars represent standard deviation (SD).


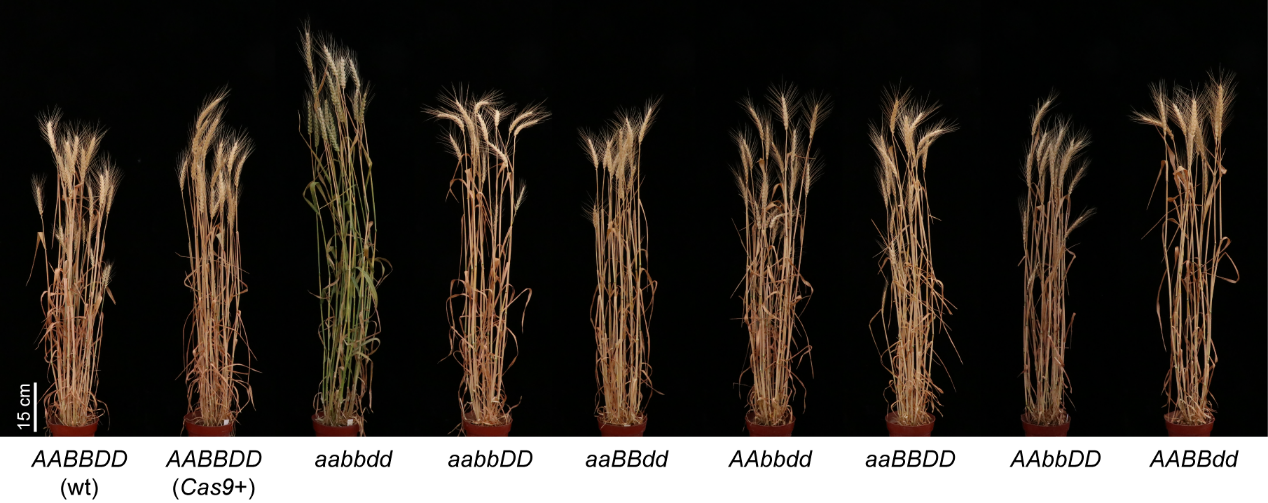


**Figure S5**. The triple knockout *TaeIF4E* mutant shows delayed maturation. The leaves and seeds of wild-type, non-edited, double-edited, and single-edited *TaeIF4E* mutant plants are dry, while the leaves and seeds of the *TaeIF4E* triple knockout mutant are light green.

1. **The three supplementary Tables for this article are as follows:**

**Table S1.** Editing events in 65 regenerated T_0_ plants, as determined by Sanger sequencing of plasmids derived from PCR products.

| **No.** | **Lines** | **A sub−genome** | | **B sub−genome** | | **D sub−genome** | |
| --- | --- | --- | --- | --- | --- | --- | --- |
|  |  | **Mutations** | **Denotation** | **Mutations** | **Denotation** | **Mutations** | **Denotation** |
| 1 | 15 | +1 bp, wt | Het | wt |  | wt |  |
| 2 | 30 | +1 bp, wt | Het | wt |  | wt |  |
| 3 | 41 | Wt |  | wt |  | −2 bp, wt | Het |
| 4 | 54 | Wt |  | wt |  | −1 bp, wt | Het |
| 5 | 87 | Wt |  | −1 bp, wt | Het | wt |  |
| 6 | 93 | Wt |  | C to A, wt | Het | wt |  |
| 7 | 103 | Wt |  | +1 bp, wt | Het | wt |  |
| 8 | 109 | −1 bp, wt | Het | wt |  | wt |  |

Het, heterozygote; wt, wild type. Lines, representing the No. of regenerated T_0_ plants.

| **Name of putative off-target site** | **Putative off-target locus** | **Sequence of the putative off-target site** | **No. of mis-matching bases** | **Mutations** |
| --- | --- | --- | --- | --- |
| off-target1 | TraesCS6A02G212000 | CCTTGCATGGACCTTCTGGTTCG | 4 | no |
| off-target2 | TraesCS6B02G242900 | CCTTGCATGGACCTTCTGGTTCG | 4 | no |
| off-target3 | TraesCS6D02G195600 | CCTTGCATGGACCTTCTGGTTTG | 5 | no |
| off-target4 | TraesCS7A02G182000LC | TCTCCTTCTGGTTCGACAACTGG | 3 | no |
| off-target5 | TraesCS3B02G739400LC | TTTCCTTCTGGTTCGACAACTGG | 3 | no |

**Table S2.** Analysis of potential off-target effects in edited mutants.

The PAM motifs are underlined and highlighted in blue, mismatching bases are shown in red.

**Table S3.** PCR primers used in this study.

| Name | Sequence (5' to 3') | Experimental purpose | Source |
| --- | --- | --- | --- |
| TaeIF4E-ABD-F | CGAAGGCGAGACCAGACCTCAC | Target all three *TaeIF4E* alleles | This study |
| TaeIF4E-ABD-R | AGAAGTCCTCGACGGTGGAGAAG |  | This study |
| TaeIF4E_A-F | GCTTCGATAGGCTTTGCTTCTG | Amplify *TaeIF4E* A allele | This study |
| TaeIF4E_A-R | CGTGTCCATCAAATAATCATGGTG |  | This study |
| TaeIF4E_B-F | CTGCGGGTGCGTCAGCTTAAT | Amplify *TaeIF4E* B allele | This study |
| TaeIF4E_B-R | AAAACTGCACGACGATTTCAC |  | This study |
| TaeIF4E_D-F | GCCGGCACGATAGTAGTAATAATCG | Amplify *TaeIF4E* D allele | This study |
| TaeIF4E_D-R | CCACATCAAGTATTTTCAGAAAAACCTTCC |  | This study |
| dCAPs_*Apa* I-F | CCCACCCGCTCGAGAACGCCTGGGCC | Target 1-bp insertion at A allele, digestion by *Apa* I | This study |
| dCAPs_*Sca* I-F | TCAGCGCCCACCCGCTGGAGAACGCCAGTAC | Target 1-bp deletion at B allele, digestion by *Sca* I | This study |
| dCAPs-R | AGAAGTCCTCGACGGTGGAGAAGGTG | dCAPs reverse primer | This study |
| Cas9-F | TCAAGGCTCTTGTTCGTCAGCA | Detect CRISPR/Cas9 cassette | Kan et al. 2022 |
| Cas9-R | TTGCCGCTCTGCTTATCCCTGA |  | Kan et al. 2022 |
| WYMV qPCR F | GACAAATTAAAGAGCGCACCCA | qRT-PCR of WYMV accumulation | Kan et al. 2022 |
| WYMV qPCR R | TAGCGTGAACAATGAATGGGGA |  | Kan et al. 2022 |
| WYMV RT F | CCGCCACCAAAGAGAAATGG | RT-PCR of WYMV accumulation | Kan et al. 2022 |
| WYMV RT R | TCGGAGGTGAGCATGGTATT |  | Kan et al. 2022 |
| CWMV qPCR F | AGTTGAGACATGGCAGAGTACG | qRT-PCR of CWMV accumulation | Kan et al. 2022 |
| CWMV qPCR R | TCTGTTTCTATCTGGCCAGCAG |  | Kan et al. 2022 |
| CWMV RT F | GGCCGTGAAATCTGGTTATACTG | RT-PCR of CWMV accumulation | This study |
| CWMV RT R | GTCTGCCCTTGTTCTTCTGTTTC |  | This study |
| Taactin F | GTGTGATGTGGATATCAGGAAGG | Endogenous control (*Actin* gene) | Kan et al. 2022 |
| Taactin R | TTAGAAGCACTTCCGGTGGA |  | Kan et al. 2022 |
| off-target1 F | CCATTCGCCTATATAAATACTCCTA | Detection mutations on putative off-target site of TaeIF4E target | This study |
| off-target1 R | CAGGGTGCTGTCGCGGGCCTT |  | This study |
| off-target2 F | GATCATGCTCGATCGTTTTACC | Detection mutations on putative off-target site of TaeIF4E target | This study |
| off-target2 R | GCGAATGGTGTTCGATTGCAGA |  | This study |
| off-target3 F | GATCATGCATTCATGCTCGAT | Detection mutations on putative off-target site of TaeIF4E target | This study |
| off-target3 R | CTTCGGTCGACTGTTTGGCATT |  | This study |
| off-target4 F | GTGCACTAGATGCCATGAGAAGA | Detection mutations on putative off-target site of TaeIF4E target | This study |
| off-target4 R | CAAGCCACCAAACCTCATAAGTCG |  | This study |
| off-target5 F | TGGAGTGCAGCTCAGGGGGGC | Detection mutations on putative off-target site of TaeIF4E target | This study |
| off-target5 R | CCGCCAAACCGCAGCAACTT |  | This study |

1. **Supplementary Methods**

**Plant materials and bacterial strains**

Hexaploid common wheat (*Triticum aestivum* L.) cv. ‘Fielder’ was used in this study as the receptor genotype. Fielder is an American soft white, pastry-type wheat variety that was released in 1974 and is well-known for its amenability to *Agrobacterium tumefaciens*-mediated transformation and genome editing (Ishida et al., 2015). The seeds of wild-type Fielder were kindly provided by Prof. Xingguo Ye from the Institute of Crop Sciences, Chinese Academy of Agricultural Sciences (CAAS). All plants were grown in soil-filled plastic pots in a growth chamber maintained at 24°C, 16-h light/18°C, 8-h dark with 300 μmol/m^2^/s light intensity at 45% relative humidity. *Agrobacterium tumefaciens* strain C58C1 and an *Escherichia coli* strain containing the helper plasmid pRK2013 were kindly provided by Prof. Xingguo Ye as well.

**Construction of the CRISPR/Cas9 vectors for gene editing**

The highly specific target sequences (Stein et al., 2005) were designed using the web-based tool CRISPR-P 2.0 (Liu et al., 2017). The CRISPR/Cas9 constructs were based on the vector pWMB110-*SpCas9,* which contains the maize *Ubiquitin* promoter to drive the expression of *Cas9* and the *bar* gene as a selection marker to identify transgenic plants (Liu et al., 2020). The sgRNA expression cassette driven by the wheat *U3* promoter was introduced into pWMB110-*SpCas9* using an In-Fusion cloning kit (Takara Bio Inc, Japan), followed by transformation into Agrobacterium strain C58C1 by triparental mating (Ditta et al., 1980). All primers used in this study are listed in **Table S3**.

**Agrobacterium*-*mediated wheat transformation**

Immature wild-type Fielder embryos were isolated at 14 days post-anthesis for Agrobacterium-mediated transformation (Wang et al., 2017). In detail, the surfaces of developing grains were sterilized with sodium hypochlorite. Immature embryos were isolated from the grains and co-cultivated with a liquid culture of Agrobacterium harboring the CRISPR/Cas9 vectors at 23°C for 2 days in the dark. The entire embryo axis was excised from each embryo using a scalpel and forceps and cultured at 25°C for 5 days in the dark with the scutellum side up on medium containing 250 mg/L carbenicillin (PhytoTech, Lenexa, KS, USA) and 100 mg/L cefotaxime (Amresco Inc, USA), followed by culture on callus induction medium containing 10 mg/L phosphinothricin (Sigma-Aldrich, St Louis, USA) to select transgenic plants containing an integrated *bar* gene. Proliferated calli were transferred to regeneration medium and cultured for 2 weeks at 25°C under constant illumination at 100 μmol/m^2^/s. The regenerated shoots were transferred onto rooting medium, and seedlings with well-developed roots were transplanted to pots containing soil for further growth in a growth chamber until seed harvest.

**DNA extraction, genotyping, and Sanger sequencing of target fragments**

Genomic DNA extraction, quantification, and quality control were conducted as previously described (Shi et al., 2019). Genome-edited sites were identified by restriction endonuclease digestion and validated by Sanger sequencing. For T_0_ generation plants, a universal primer set that amplifies all three homoeoalleles of *TaEIF4E* was used to identify any possibly edited loci. The purified PCR products were subcloned into sequencing plasmids, and 8 to 10 positive colonies were selected and subjected to Sanger sequencing. To genotype T_1_ segregants and their progeny, nested PCR was performed. Briefly, the first round of PCR was conducted using subgenome-specific primers to amplify the corresponding fragments, which then served as the template for the second round of PCR using target-specific primers (Li et al., 2018; Neff et al., 1998) to generate the derived cleaved amplified polymorphic sequence (dCAPS) marker. The A and B subgenome-specific dCAPS primers were designed using dCAPS Finder 2.0 (Neff et al., 2002). However, no suitable primers or restriction endonucleases were available for the D subgenome. Therefore, the editing site was identified by Sanger sequencing of the first-round PCR products. The edited plants that were identified with the dCAPS marker were further validated by Sanger sequencing. All primers and restriction endonucleases used in this study are listed in **Table S3**.

**Pyramiding and selection of double- and triple-edited lines**

Two rounds of cross-pollination and marker-assisted selection were conducted to pyramid the *TaeIF4E* frameshift mutation from each subgenome (A: 1-bp insertion; B: 1-bp deletion; D: 2-bp deletion) into a single plant. In detail, homozygous T_1_ plants #15 (A subgenome: 1-bp insertion) containing an edited *TaeIF4E* gene in a single subgenome were cross-pollinated with other T_1_ plants #41 (D subgenome: 2-bp deletion) to develop edited heterozygotes in two subgenomes. The resulting T_2_ hybrids (*AaBBDd*) were cross-pollinated with homozygous plants #87 (B subgenome: 1-bp deletion, *AAbbDD*) carrying an edited *TaeIF4E* gene in the third subgenome. The heterozygous triple-edited plants (*AaBbDd)* were self-pollinated to develop a segregating population containing all eight haplotype combinations for the three homeoalleles. Among the segregants, single-, double-, and triple-edited mutants of *TaeIF4E* were identified by marker-assisted genotyping, followed by validation by Sanger sequencing. Plants of various genotypes were retained for seed setting and bulking. Embryo culture was performed to accelerate seed multiplication (Abe et al., 2019).

**Detection of the off-target events**

To detect the off-target events, the target site was checked through BLASTN against the reference genome sequence of wheat (IWGSC RefSeq v1.1) (Consortium, 2018), and five putative off-target sites showing levels of similarity to the target sequence were predicted. Sequence-specific PCR amplification and Sanger sequencing was used to determine the potential off-target effects in edited mutants. The primer sets were listed in **Table S3**.

**Evaluation of virus resistance using mechanical inoculation**

Disease resistance against WYMV and CWMV was evaluated by mechanical inoculation in a growth chamber under a 12°C, 10-h day/8°C, 14-h night cycle (Shi et al., 2019). WYMV-infected wheat leaves were collected from Yangzhou city in Jiangsu Province, China, and CWMV-infected leaves were collected from Linyi city in Shandong Province, China. Two-week-old wheat seedlings were mechanically inoculated twice at an interval of 5 days using leaf sap from virus-infected leaves mixed with K_2_HPO_4_ buffer (1:10; 0.1 M; pH 9.1) and silicon carbide (carborundum, mesh 150–200, 0.5 g/25 ml sap). Mock inoculation was performed using K_2_HPO_4_ buffer and silicon carbide only. For each genotype, fifteen plants were inoculated with infectious leaf sap and five other plants were mock-inoculated in each experiment; two independent experiments were conducted. Five weeks after the first inoculation, the youngest leaf of each plant was collected for total RNA extraction using TRIzol reagent (Invitrogen, USA), followed by first-strand cDNA synthesis using HiScript III RT SuperMix (Vazyme Biotech, Nanjing, China). Quantitative PCR (qPCR) was performed with a ChamQ Universal SYBR qPCR Master mix (Vazyme Biotech, Nanjing, China) on an ABI 7500 Real-Time PCR system (Applied Biosystems, USA). The wheat *Actin* gene was used as an endogenous control (Kan et al., 2022), and relative gene expression was calculated using the 2^–ΔΔCT^ method (Livak and Schmittgen, 2001). The specific primers used to amplify the genomic fragment of WYMV or CWMV, as well as the *Actin* gene, are listed in **Table S3**.

**Evaluation of agronomic performance under infectious and normal conditions**

After evaluating virus resistance, all WYMV-inoculated and mock-inoculated plants of each genotype were transplanted into pots containing soil and grown in a greenhouse until harvest to evaluate plant height and seed setting. In addition, seeds of different genotypes were sown in pots containing soil and grown under bymovirus-free conditions to evaluate their agronomic performance throughout the wheat growing season beginning in the spring of 2022. Twelve agronomic traits were evaluated: plant height (PH), days to heading (HD), days to maturation (MD), tiller number (TL), spike number per plant (SNP), grain number per main spike (GNS), spike length (SL), grain weight per plant (GWP), grain length (GL), grain width (GW), the ratio of grain length to width (RLW), and thousand-grain weight (TGW). For each trait, 10 to 15 plants per genotype were analyzed. GL, GW, RLW, and TGW were measured with a WSeen measuring system (SC-G, WSeen Detection Tech, Hangzhou, China).

**References**

Abe, F., Haque, E., Hisano, H., Tanaka, T., Kamiya, Y., Mikami, M., Kawaura, K., Endo, M., Onishi, K., Hayashi, T. and Sato, K. (2019) Genome-edited triple-recessive mutation alters seed dormancy in wheat. *Cell Reports* **28**, 1362-1369.

Chandrasekaran, J., Brumin, M., Wolf, D., Leibman, D., Klap, C., Pearlsman, M., Sherman, A., Arazi, T. and Gal-On, A. (2016) Development of broad virus resistance in non-transgenic cucumber using CRISPR/Cas9 technology. *Molecular Plant Pathology* **17**, 1140-1153.

Consortium, I.W.G.S. (2018) Shifting the limits in wheat research and breeding using a fully annotated reference genome. *Science* **361**, eaar7191.

Ditta, G., Stanfield, S., Corbin, D. and Helinski, D.R. (1980) Broad host range DNA cloning system for gram-negative bacteria: construction of a gene bank of *Rhizobium meliloti*. *Proceedings of The National Academy of Sciences of the United States of America* **77**, 7347-7351.

Ishida, Y., Tsunashima, M., Hiei, Y. and Komari, T. (2015) Wheat (*Triticum aestivum* L.) transformation using immature embryos. In: *Methods in Molecular Biology* (Wang, K. ed), pp 189-198. New York: Springer.

Jackson, R.J., Hellen, C.U. and Pestova, T.V. (2010) The mechanism of eukaryotic translation initiation and principles of its regulation. *Nature Reviews Molecular Cell Biology* **11**, 113-127.

Kan, J.H., Cai, Y., Cheng, C.Y., Jiang, C.C., Jin, Y.L. and Yang, P. (2022) Simultaneous editing of host factor gene *TaPDIL5-1* homoeoalleles confers wheat yellow mosaic virus resistance in hexaploid wheat. *New Phytologist* **234**, 340-344.

Li, L., Liu, J., Xue, X., Li, C., Yang, Z. and Li, T. (2018) CAPS/dCAPS Designer: a web-based high-throughput dCAPS marker design tool. *Science China Life Sciences* **61**, 992-995.

Liu, H., Ding, Y.D., Zhou, Y.Q., Jin, W.Q., Xie, K.B. and Chen, L.L. (2017) CRISPR-P 2.0: An Improved CRISPR-Cas9 tool for genome editing in plants. *Molecular Plant* **10**, 530-532.

Liu, H.Y., Wang, K., Jia, Z.M., Gong, Q., Lin, Z.S., Du, L.P., Pei, X.W. and Ye, X.G. (2020) Efficient induction of haploid plants in wheat by editing of *TaMTL* using an optimized *Agrobacterium*-mediated CRISPR system. *Journal of Experimental Botany* **71**, 1337-1349.

Livak, K.J. and Schmittgen, T.D. (2001) Analysis of relative gene expression data using real-time quantitative PCR and the 2^−ΔΔCT^ method. *Methods* **25**, 402-408.

Neff, M.M., Neff, J.D., Chory, J. and Pepper, A.E. (1998) dCAPS, a simple technique for the genetic analysis of single nucleotide polymorphisms: experimental applications in *Arabidopsis thaliana* genetics. *Plant Journal* **14**, 387-392.

Neff, M.M., Turk, E. and Kalishman, M. (2002) Web-based primer design for single nucleotide polymorphism analysis. *Trends in Genetics* **18**, 613-615.

Shi, L., Jiang, C., He, Q., Habekuss, A., Ordon, F., Luan, H., Shen, H., Liu, J., Feng, Z., Zhang, J. and Yang, P. (2019) Bulked segregant RNA-sequencing (BSR-seq) identified a novel rare allele of *eIF4E* effective against multiple isolates of BaYMV/BaMMV. *Theoretical and Applied Genetics* **132**, 1777-1788.

Stein, N., Perovic, D., Kumlehn, J., Pellio, B., Stracke, S., Streng, S., Ordon, F. and Graner, A. (2005) The eukaryotic translation initiation factor 4E confers multiallelic recessive *Bymovirus* resistance in *Hordeum vulgare* (L.). *Plant Journal* **42**, 912-922.

Wang, K., Liu, H.Y., Du, L.P. and Ye, X.G. (2017) Generation of marker-free transgenic hexaploid wheat via an *Agrobacterium*-mediated co-transformation strategy in commercial Chinese wheat varieties. *Plant Biotechnology Journal* **15**, 614-623.

Yoon, Y.J., Venkatesh, J., Lee, J.H., Kim, J., Lee, H.E., Kim, D.S. and Kang, B.C. (2020) Genome editing of *eIF4E1* in tomato confers resistance to *Pepper mottle virus*. *Frontiers in Plant Science* **11**, 1098.
