# Supplementary figures and images for: CRISPR/Cas9‐guided knockout of eIF4E improves Wheat yellow mosaic virus resistance without yield penalty
Source: Plant Biotechnol J. 2023 Jan 24;21(5):893–5. doi: 10.1111/pbi.14002 (PMC10106853; doi:10.1111/pbi.14002)

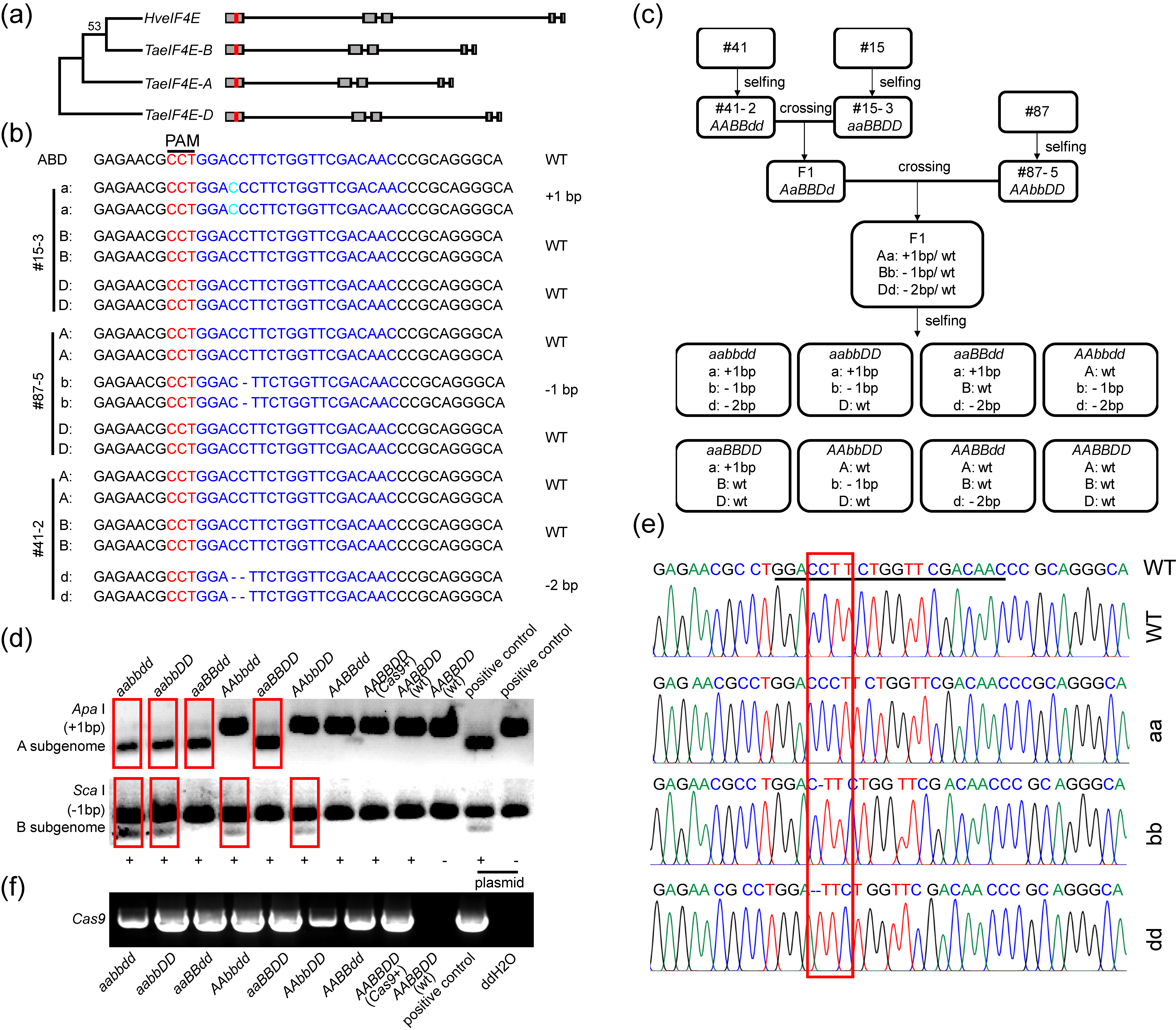

Supplement: Supplementary file 2 — Figure S1. Generation of TaeIF4E‐edited mutants by genome editing and marker‐assisted allele stacking. [file PBI-21-893-s005.tif]

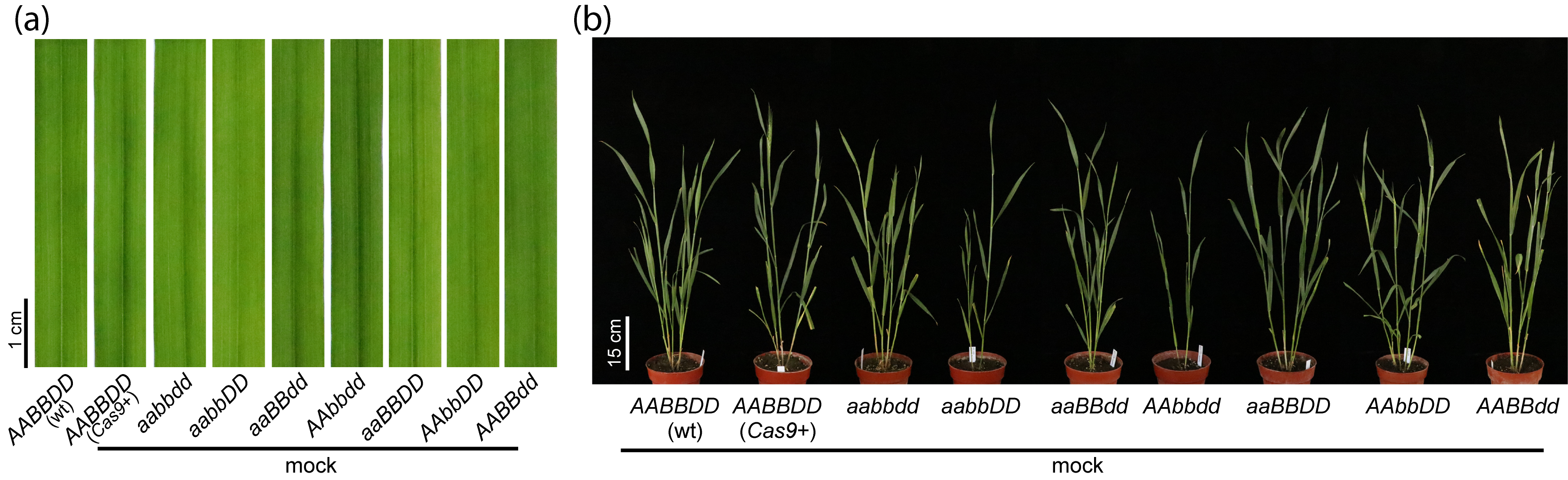

Supplement: Supplementary file 3 — Figure S2. Phenotypes of the TaeIF4E‐edited mutant lines following mock inoculation under greenhouse conditions. [file PBI-21-893-s004.tif]

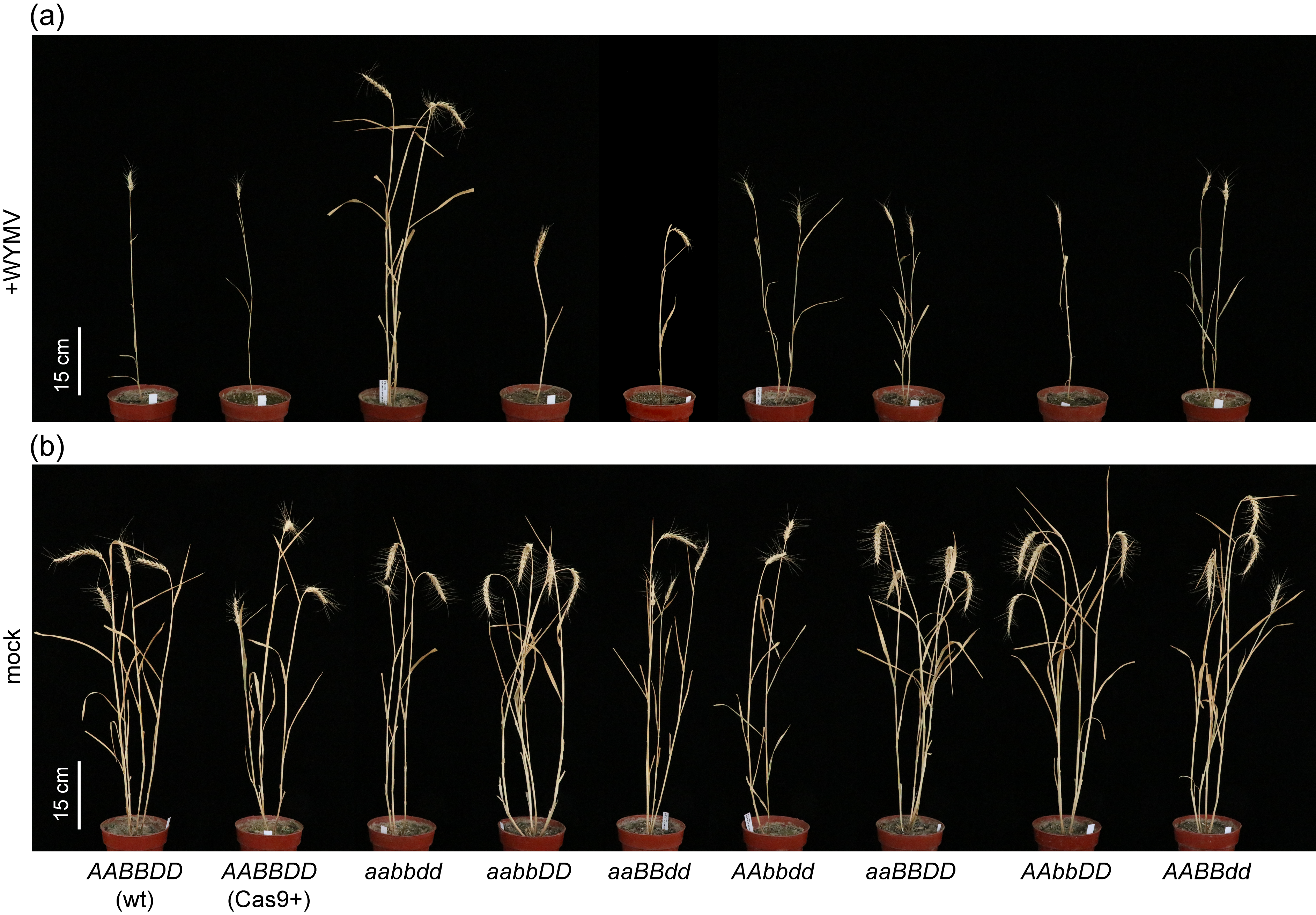

Supplement: Supplementary file 4 — Figure S3. Phenotypes of WYMV‐inoculated and mock‐inoculated plants at the maturation stage under greenhouse conditions. [file PBI-21-893-s002.tif]

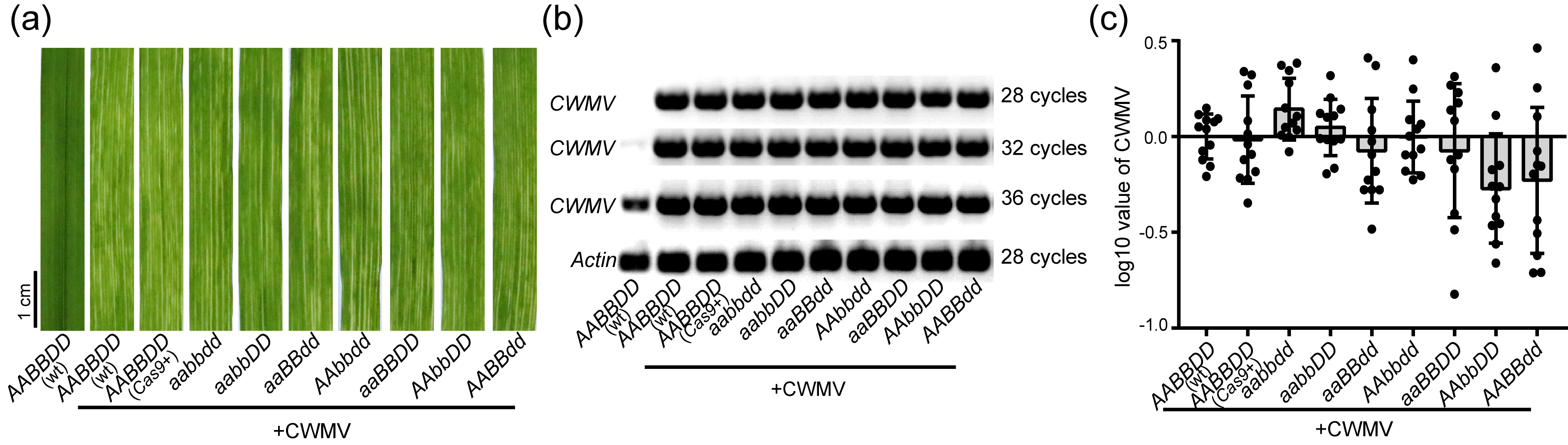

Supplement: Supplementary file 5 — Figure S4. TaeIF4E‐edited triple mutant in common wheat shows no resistance to CWMV. [file PBI-21-893-s001.tif]

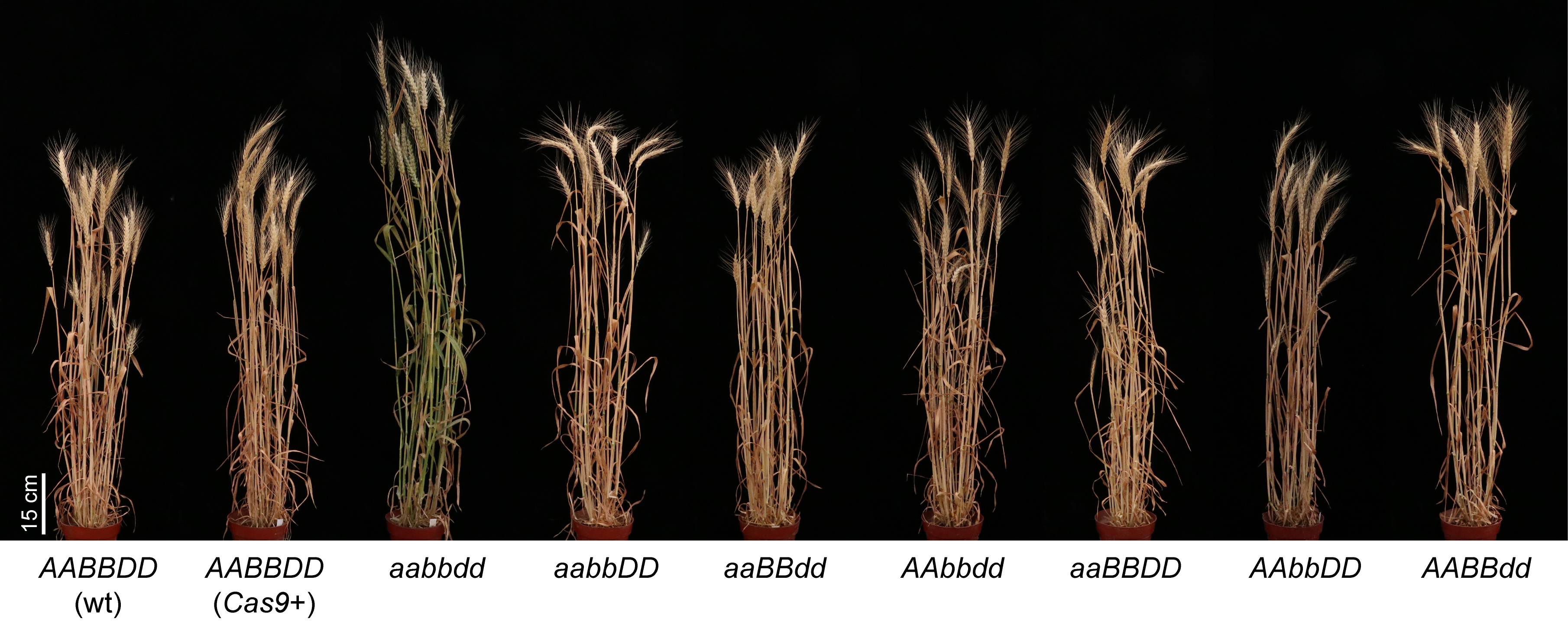

Supplement: Supplementary file 6 — Figure S5. Triple knockout TaeIF4E mutant shows delayed maturation. [file PBI-21-893-s006.tif]
